# Do it together! Conception and long-term results of the trans-institutional Master of Medical Education (MME) program in Germany

**DOI:** 10.3205/zma001326

**Published:** 2020-04-15

**Authors:** Jana Jünger, Saskia V. Pante, Kristina Ackel-Eisnach, Stefan Wagener, Martin R. Fischer

**Affiliations:** 1 Heidelberg University, Medical Faculty of Heidelberg, MME program, Heidelberg, Germany; 2 Institute of Medical and Pharmaceutical Proficiency Assessment, Mainz, Germany; 3 University of Koblenz/Landau, Empirical Pedagogical Research, Landau, Germany; 4 University Hospital, LMU Munich, Institute for Medical Education, Munich, Germany

**Keywords:** Master program, medical education, model character, postgraduate, interfaculty, train-the-trainer, evaluation

## Abstract

Medical education has the responsibility to react to developments and changing demands in healthcare. This implies the need for experts in the area of medical education as well as nationally coordinated initiatives. An innovative model based on trans-institutional cooperation and nationwide consensus for establishing a master’s degree course in Medical Education (MME) and long-term results are presented here to other countries and other programs, facing similar challenges. A MME program with the following goals was implemented at the Medical Faculty of Heidelberg University, Germany, in 2004:

1. Qualification of leaders in medical faculties,
2. professionalization and improvement of teaching quality,
3. promotion of nationwide and international exchange, and
4. stimulation of research in medical education.

Since then, 15 cohorts with a total of 380 participants have started their studies, 179 participants have graduated and 90 publications resulted from the master’s theses (as at November 2018). Evaluation and survey data revealed a very high degree of satisfaction among the participants and a lasting development to medical education experts. Our concept shows that the bundling of regional expertise into a clearly structured trans-institutional network can be a driving force for nationwide comprehensive changes, in order to address changing demands in healthcare systems and transfer it into medical education programs.

## Zusammenfassung

Die medizinische Ausbildung ist dazu verpflichtet, auf Entwicklungen und veränderte Anforderungen im Gesundheitswesen zu reagieren. Diese Prozesse benötigen Experten auf dem Gebiet der medizinischen Ausbildung sowie national koordinierte Initiativen. Ein innovatives Modell, das auf institutsübergreifender Zusammenarbeit und landesweitem Konsens für die Einrichtung eines Master-Studiengangs in Medizinischer Ausbildung (MME) basiert, wird hier zusammen mit langfristigen Ergebnissen anderen Ländern und Programmen vorgestellt, die vor ähnlichen Herausforderungen stehen. An der Medizinischen Fakultät der Universität Heidelberg in Deutschland wurde 2004 ein MME-Programm mit den folgenden Zielen eingeführt:

1. Qualifizierung von Ausbildungstrainern und Multiplikatoren sowie Führungspersonen insbesondere aus der Hochschulmedizin,
2. Professionalisierung und Erhöhung der Qualität der Medizinischen Ausbildung,
3. Förderung des bundesweiten und internationalen wissenschaftlichen Austausches und
4. Verbesserung der Ausbildungsforschung.

Seitdem haben 15 Kohorten mit insgesamt 380 Teilnehmern ihr Studium begonnen, 179 Teilnehmer haben ihr Studium abgeschlossen und 90 Publikationen sind aus den Masterarbeiten hervorgegangen (Stand: November 2018). Evaluationen und Umfragen zeigten eine sehr hohe Zufriedenheit unter den Teilnehmern und eine nachhaltige Entwicklung zu medizinischen Ausbildungsexperten. Unser Konzept belegt, dass die Bündelung von regionaler Expertise in einem klar strukturierten, trans-institutionellen Netzwerk eine treibende Kraft für bundesweite, umfassende Neugestaltungen sein kann, um den veränderten Anforderungen im Gesundheitswesen gerecht zu werden und diese in medizinische Ausbildungsprogramme zu übertragen.

## Background

In order to meet the various tasks and requirements of the medical profession, a continuous further development of the medical education including changing demands in healthcare is essential [1]. Over the past two decades the need for medical experts who develop new curricula and teaching methods and transfer findings from educational research to medical education has grown significantly [2], [3], [4]. At the same time, the number of qualification programs for teaching staff, especially those which are locally based, has been rising globally since 1990 [5], [6], [7], [8]. A report on master’s degrees in medical education identified a rise from 15 to 21 programs in the English-speaking world in 1998 [3], [9] to 76 in 2012 [10] and to 121 in 2013 [11]. Supporting this positive trend, presently, the Faimer Institute lists a number of 132 worldwide master’s Programs in Health Professions Education [https://www.faimer.org/resources/mastersmeded.html].

In a systematic review of available master’s programs in health professions/medical education worldwide Tekian and Harris recently showed that there was a geographic maldistribution of such programs [10]. The US (15 programs) and the UK (20 programs) are clearly ahead with the largest number of programs. This reflects the long tradition of medical research in education as well as experience in the area of faculty development in the US and in the UK [12], [13], [14]. 

A major milestone for significant change in medical education in the US was the Flexner Report that revealed inadequate conditions in medical schools throughout the United States [15]. In Germany, after a devastating exodus and loss of academic excellence and culture due to the totalitarian Nazi regime and World War II, there have been few changes and reform efforts in medical education in post war West Germany. It was only in the 1990s that a broad reflection on the view of physicians and the necessary changes in medical education took place, which finally led to a new Legal Framework for Regulating Licenses to Practice Medicine (Ärztliche Approbationsordnung) in 2003 [https://www.gesetze-im-internet.de/_appro_2002/BJNR240500002.html]. It calls for stronger practical relevance in university studies, stronger interdisciplinary and case-based teaching, more bedside teaching, mandatory evaluation of teaching, a reorganization of university courses, the integration of interactive teaching methods, teaching in small groups, a reorganization of exam regulations and graded exams in all subjects at the faculty.

The coming into force of this new framework in 2003 posed an enormous challenge for all 37 German medical faculties in regard to curriculum changes. Many faculties initiated reform processes of varying degrees. Moreover, a heterogeneous range of qualifications as well as associations and funding opportunities for medical education arose in Germany over the last years [16]. 

However, there was a risk of putting a lot of effort into developing new, localized and disconnected solutions for general problems. Thus, Germany had to choose between importing extensive expertise from the outside or creating national structures and networks through the use and further development of regional expertise. To our knowledge, there is no model for a nationwide MME program world-wide so far, which addresses the national challenges and the specific regional needs embedded in an international perspective. Hence, this publication provides data about conception and long-term results of the German MME program as an example for such a national initiative.

## Program development

Following an initiative of the German Association of Medical Faculties (“Medizinischer Fakultätentag” (MFT)), an interdisciplinary model was developed which serves as a nationwide qualification program for teaching staff in medicine and regionally expertise becomes available for all faculties [17], [18]. An interfaculty committee developed a basic concept for the course Master of Medical Education (MME) with the following goals:

1. qualification of leaders in medical faculties,
2. improvement of teaching quality,
3. promotion of nationwide and international exchange and
4. fostering of research in medical education

which should altogether move professionalization of medical education in Germany forward. The committee identified specific strengths of the curricula of individual German and Austrian faculties which should be integrated in the program and named faculties who stood out due to their high expertise in the respective area. In a convent in May 2004, all 30 teaching faculties worked out the final version of the program’s curriculum due to Kern’s cycle [19]. Since then, current results of medical education research and learning theories [20] are regularly taken into account and included into the curriculum. This way, a homogenous structure and a broad consensus for the curriculum emerged. 

## Organization of the course

The MFT is patron for the German MME course, which is administered and managed at Heidelberg University. The course’s steering team consists of four members. Presently, there are seven medical faculties (Aachen, Berlin, Dresden, Göttingen, Heidelberg, Tübingen and LMU Munich) which are directly involved in organizing the attendance modules, and which entered into a mutual cooperation agreement. This agreement defined the joint implementation of the MME. This includes the organization of the modules and the agreed framework for dealing with all issues regarding the course. In a joint committee, the representatives of these seven faculties settle all general questions of the MME course under the chairmanship of a MFT representative. In addition, at least one student of the course is a member of the committee. 

In the beginning, the course was supported financially by the Stifterverband für die Deutsche Wissenschaft (Donors' Association for the Promotion of Sciences and Humanities in Germany) and the Heinz Nixdorf Foundation in the form of 72 grants which covered half of the tuition fee for two participants per faculty.

## Curriculum

At present, the seven medical faculties carry out one kick-off-module and eight one-week attendance modules of the MME. The eighth module takes place as a study trip at a renowned university in Germany. The faculties can apply for this visit of the MME participants, who will carry out a systematic evaluation of the new established medicine program of this university.

An additional ninth facultative module which addresses current trends in medical education is offered once a year to all MME participants and graduates and all MME faculty.

The modules are spread over the two years of studies. Principles of the curriculum are:

- Collaborative curriculum development
- Interfaculty organization
- Presenting contents of teaching including multiple perspectives
- Taking up and deepening contents in progressive learning phases
- Longitudinal interconnection of the modules

A fundamental element of the MME curriculum is a logical vertical organization of contents (see table 1 (Tab. 1)). The modules and the forms of teaching and learning are 

sequential in terms of methodology and didactics and develop from module 1 to 8. Additionally, a longitudinal module connects the individual modules and ensures interlocking of contents during the course. This includes a mentoring program and the constant supervision during project work and master’s theses across all modules. Overarching key concepts and topics across module are theoretical principles and model of learning, a broad variety of teaching and assessment methods. Aspects of faculty development and leadership skills are general important topics with multiple representations in the curriculum. Finally, educational scholarship is a key concept in all modules mounting in exemplary scientific work in a project report and the master's thesis. 

Project work is another important component of the curriculum and is planned as a teaching project. It aims at displaying the local visibility of participants at the faculty on the one hand and providing the faculty which is sending the participant with added value on the other hand. The master’s thesis is designed as research work in which students find a solution to a chosen problem, and which is accompanied with a generalizable knowledge gain. The master's thesis follows project work and frequently builds on the results and implementation work of the respective project. Additionally, graded tasks for each module are to be accomplished. Participants earn 60 ECTS credits during their 2-year studies, each the equivalent of a 30 hour study load. While the general structure of the curriculum rendered to be successful and remained stable over the last 15 years, the structure and content within each module was frequently changed over time based on the evaluation results of participants and of the discussions among MME faculty that mirrors current trends and changes in the field of Medical Education. 

Many of the lecturers are MME graduates themselves (from MME Berne, Switzerland and from German MME course). During the eight one-week attendance modules, three teachers from different faculties form the respective module team. In doing so, multiple perspectives from different faculties and expertise backgrounds are implemented through the mix of teaching faculty. 

The international perspective with invited lecturers is strengthened by cooperation projects by networking with international experts i.e. at the annual AMEE conference through the network of international directors of medical education master's course directors and international working groups in which the lecturers are involved. Results of these cooperation projects are regularly included in the MME program and presented at international conferences. 

## Participants

Participants are selected according to qualification criteria to gain admission to the study program. One prerequisite is at least one year's experience in medical education. This ensures that the applicants have a basic qualification and are able to fulfil the requirements of this master’s program.

Since October 2004, 15 cohorts with 380 participants (23-26 per year) from all 37 German, five Austrian and one Swiss medical faculties have started their studies (as at November 2018). Of the 380 participants, 150 (39.5%) are female and 230 (60.5%) are male. The participants being on average 40.1 years old (SD=7.6 years) when starting the course. 260 (68.4%) originate from the clinical area, followed by 43 (11.3%) from the dental area, 41 (10.7%) from the pre-clinical area, 24 (6.3%) from the clinical-theoretical area and 12 (3.2%) from other areas. In terms of the academic qualifications of the participants at the beginning of the course 238 (62.6%) participants had a doctorate and 107 (28.1%) were qualified as university lecturers.

## Quality assurance

**Figure 1:**
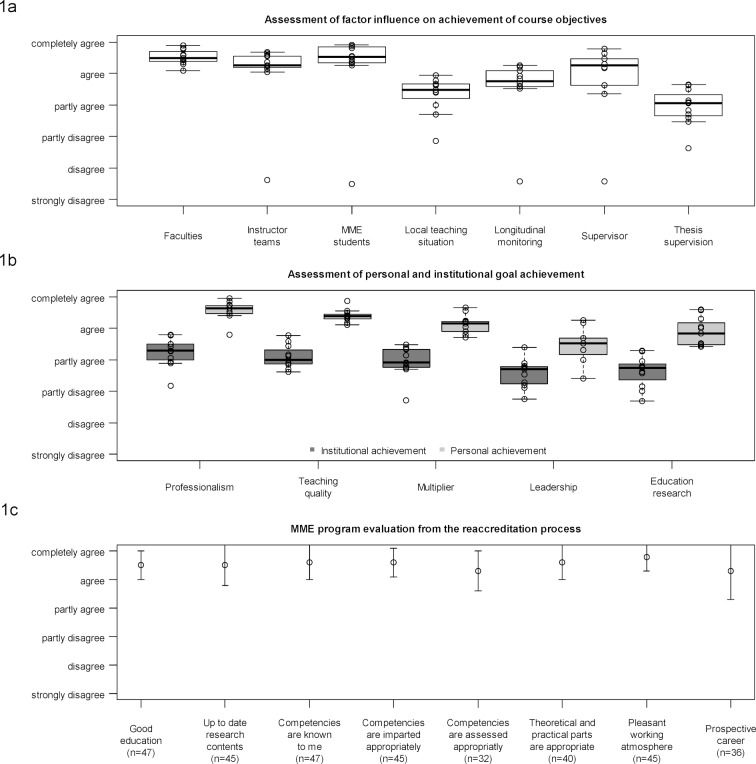
Evaluation results (a) Boxes represent the 25 and 75 percentiles, bold lines show the medians. Whiskers extend from the boxes up to +/- 1.5 times the interquartile range. Dots depict all individual observations, with those outside the boxes as well as whiskers representing outliers. Values indicate final evaluations (cohorts 2-12) on the assessment of factors that influence significantly the achievement of the course objectives (n = 181). Please note that the data was collected by the office for quality assurance at the University of Heidelberg and reported aggregated to the average value for each cohort. (b) Boxes represent the 25 and 75 percentiles, bold lines show the medians. Whiskers extend from the boxes up to +/- 1.5 times the interquartile range. Dots depict all individual observations, with those outside the boxes as well as whiskers representing outliers. Values indicate final evaluations (cohorts 2-12) on the assessment of institutional goal achievement for each faculty by the MME and personal achievement by the MME participants (n = 181). Please note that the data was collected by the office for quality assurance at the University of Heidelberg and reported aggregated to the average value for each cohort. (c) Values indicate means (as circle) and standard deviation (as error bars) of final evaluations of cohorts 10 and 11 from the reaccreditation process. Please note that the data was collected by the office for quality assurance at the University of Heidelberg and reported in anonymous and aggregated form.

The joint development of the curriculum represented the first step of quality assurance. On top of that, in each module an accompanying formative evaluation of the structure, the process and subjective learning success is carried out, complemented by a final evaluation of the whole course in the last module. This ensures constant further development of the organization and the contents of the modules, and serves the goal of ensuring high efficiency and quality of teaching. The main evaluation results and curricular changes are presented during the annual meeting of the teaching faculty. Problems across all modules are discussed and an implementation plan is worked out. Figure 1 (Fig. 1) shows the evaluation results of the final evaluation. Figure 1 (Fig. 1), point a reveals that the concept of the MME course is assessed in almost all areas as being good to very good. The improvement of personal achievements through the MME program is highly appreciated in all areas (see figure 1 (Fig. 1), point b). The institutional goal achievement is assessed as being good but not as good as personal achievements (see figure 1 (Fig. 1), point b). An additional program evaluation was performed in 2015 during the reaccreditation process. Selected results are shown in figure 1 (Fig. 1), point c. These results reveal the positive impression of the participants about the curriculum, teaching standard and working atmosphere.

To support the individual professionalization of each participant, in 2014 a specific competence-oriented roles matrix of self-evaluation based on the CanMEDS roles [21] was implemented in the MME program [22]. By this instrument, the competency development of the participants to medical education experts becomes transparent and can be reflected, documented and promoted. Importantly, this also helps to ensure quality of the MME program. For a detailed overview about the defined roles, see table 2 (Tab. 2).

## Qualitative data

At the end of the two-year-curriculum evaluation data from participants' free text comments (Cohort 10 and 11, n=44) can be summarized as follows: The curriculum is conclusive and the sequence of modules is well orchestrated. The alignment of learning goals, teaching and learning and the application and transfer into educational practice works well. Furthermore, the productive group dynamics as well as a positive learning atmosphere and the interdisciplinary multiplicity of expertise are emphasized. Suggestions for improvement focus on more educational research input, a stronger linkage across and between cohorts and a more flexible curricular structure.

## Outcomes

**Figure 2:**
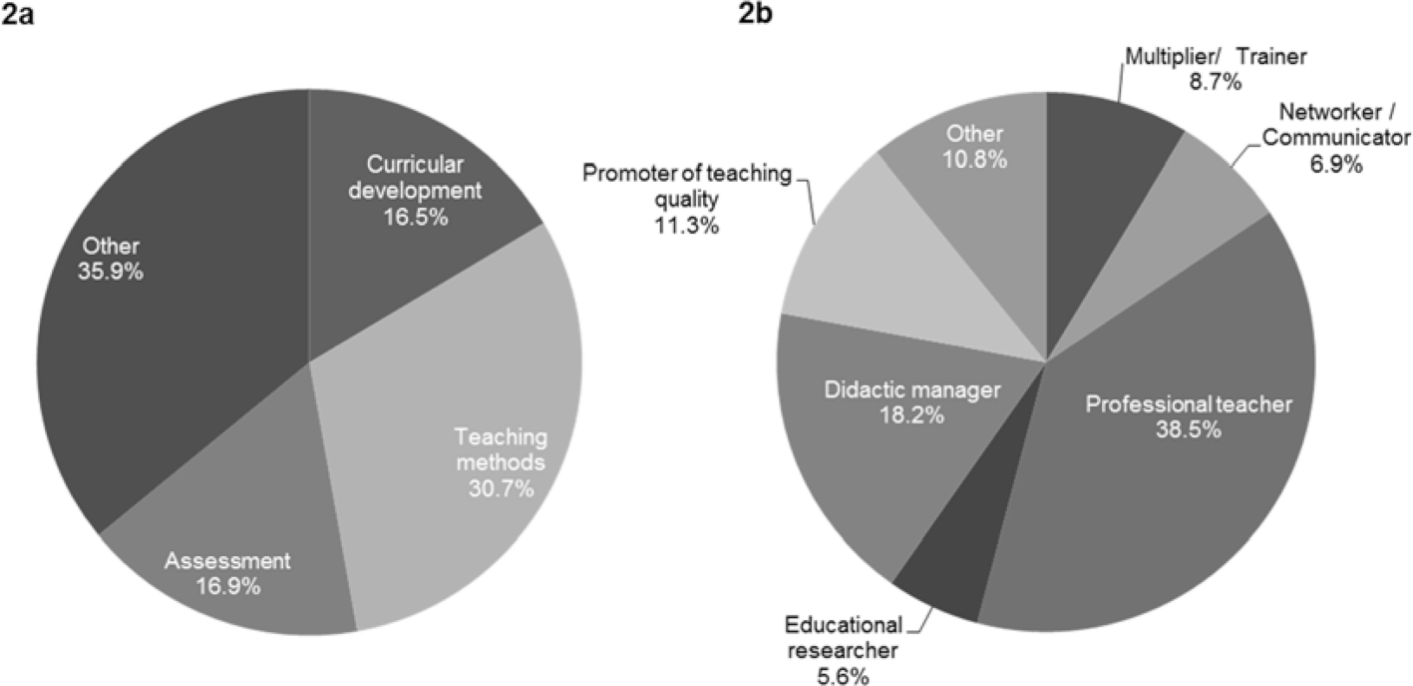
Assignments of master’s theses Assignment of master’s theses of cohorts 2-12 to (a) curricular components and to (b) the roles of the roles matrix (n=231).

So far, 179 participants have finished their studies (as at November 2018). This represents 59.3% of the participants from the first twelve years (n=302). Among the graduates, there are 109 (60.9%) men and 70 (39.1%) women. The graduates are on average 43.5 years old (SD=6.7 years). The classification of the master’s theses to the curricular components of the MME program shows that 71 (30.7%) of the completed or registered master’s theses (n=231) can be assigned to teaching methods, 39 (16.9%) to assessment and 38 to (16.5%) curricular development (see figure 2 (Fig. 2), point a).

With regard to the roles of the roles matrix 89 (38.5%) of the theses cover the role professional teacher and 42 (18.2%) of the didactic manager (see figure 2 (Fig. 2), point b). 

**Figure 3:**
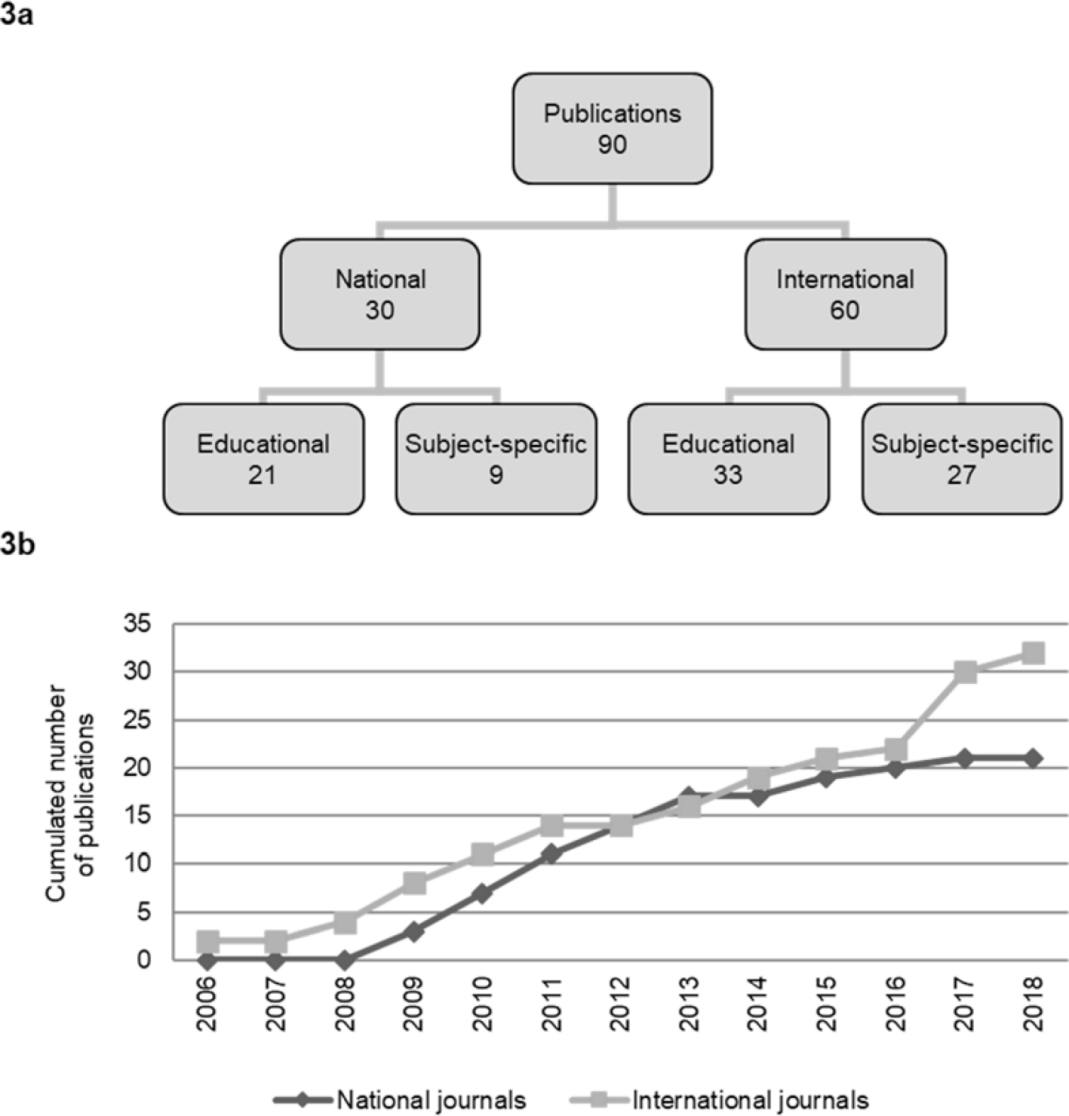
Publications resulted from MME master’s theses (a) Classification of the published MME master’s theses to journal types, (b) Cumulated number of educational publications from MME master’s theses from year 2006 to 2018.

Taking into account the three key criteria of scholarship, we encourage the participants to publish their master’s theses in a peer-reviewed journal to make it accessible to the scientific community [23]. So far, 90 publications resulted from the master’s theses and there were 33 which can be assigned to international specialized education journals (e.g. [24], [25], [26], [27]). Figure 3 (Fig. 3), point a gives a detailed overview about the publications. Figure 3 (Fig. 3), point b represents the cumulated number of educational publications resulting from MME master’s theses over the last 12 years. 

All participants of cohort 1-10 were invited to participate in a retrospective online survey in April/June 2016. 157 out of 246 (63.8%) took part in this alumni survey dealing with MME master’s theses, stress levels and career opportunities of the participants [28], [29].

**Figure 4:**
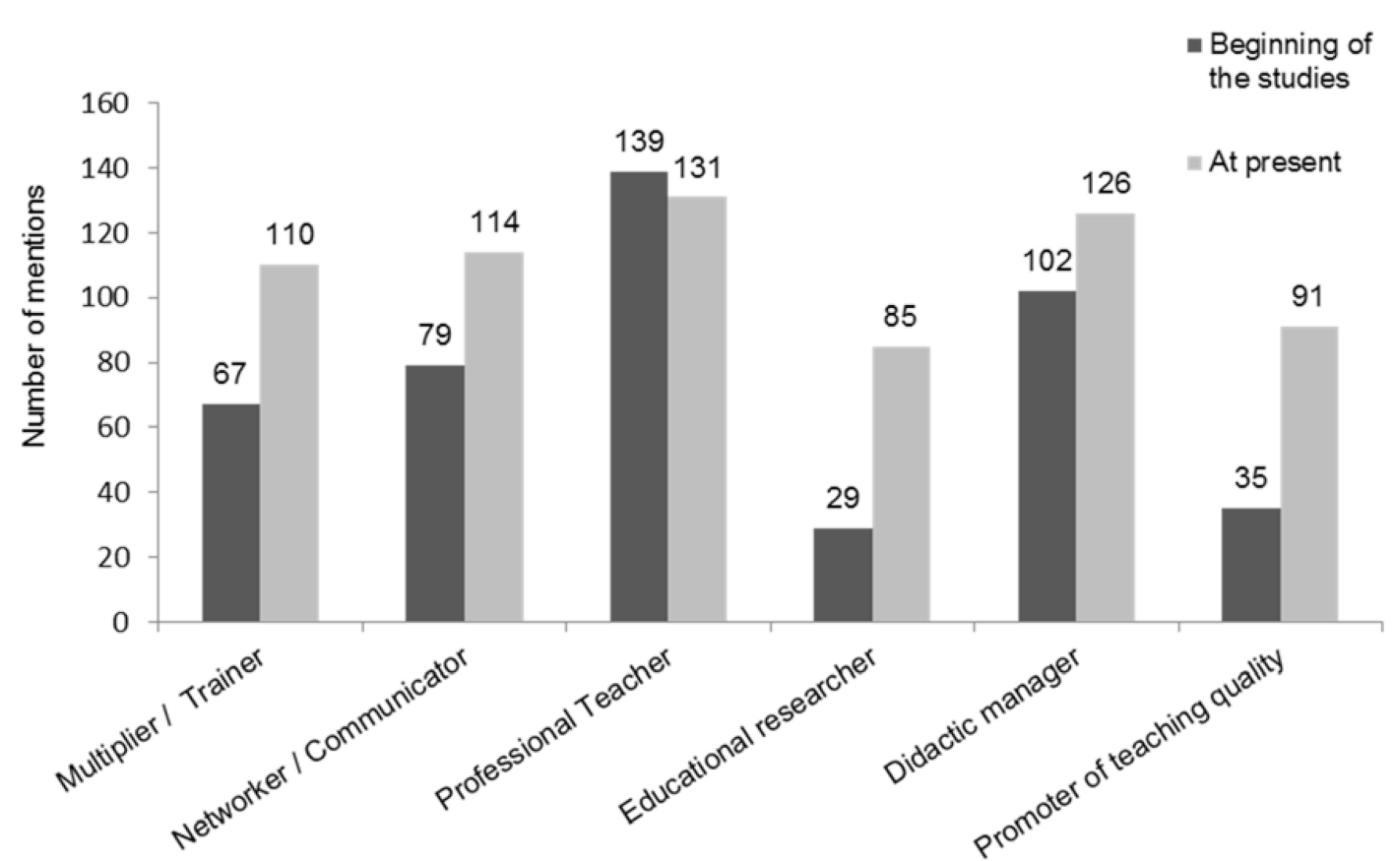
Development of medical education expertise Total number of mentions of occupied teaching roles by the participants from cohort 1-10 at the beginning of the studies and at present (multiple answers possible, n=157).

In order to analyze the development of the participants to medical education experts, the participants were asked about the fulfillment of the teaching roles of the roles matrix before attending the MME program and at present. Figure 4 (Fig. 4) reveals that the expertise in almost all roles is strengthened, especially in the ones of an educational researcher and of a promoter of teaching quality.

The MME alumni criticized that their home faculties still devote too few resources and too low support for medical education research. However, the alumni survey also revealed that successful raising third-party funds in medical education increased after the graduation of the MME program.

The number of MME graduates within the German-speaking community is rising every year and medical education experts are more and more employed in Dean's Offices in positions with decision and change commitment. In addition, MME graduates are significantly involved in national and international research projects on education research. Four years after starting the program, a MME graduate from the University of Berne was holding the first chair in medical education at a German university. To date, 8 out of 9 chairs are currently staffed by MME graduates. 

## Discussion

The MME course of the MFT at Heidelberg University is successfully implemented as a postgraduate, interfaculty academic education program for promoting the increase of professionalism in medical teaching. The implementation of the course in originally eight different locations and close interfaculty cooperation and networking represents a structural innovation. 

If one compares the elements of the course with the recommendations of the BEME Guide No. 8 for the development of faculty development programs [7], some aspects will become more obvious than others: 40% of the programs aim at interdisciplinary organization but only 10% train clinicians and pre-clinicians together. It is exactly this, however, that has proven to be one of the strengths of the German MME curriculum. Through discussion about learning objectives, understanding for the other curricular objectives is created, and the widely spread overestimation of one’s own professional area is relativized. 

To evaluate program outcomes beyond participant satisfaction, Kirkpatrick’s levels of evaluation are used [30]. Participant satisfaction (Kirkpatrick’s first level) could be confirmed by the program evaluation. Central basic concepts of the course such as the interfaculty organization and composition of lecturer teams, the great importance of forming groups and networking as well as longitudinal supervision are very much appreciated by participants. Forming of groups and networking have already proven to be crucial for the learning success [31], [32]. The organization of attendance modules in different locations, each of which follow other paths in the professionalization of their teaching, prevents participants from searching for a “single perfect intervention” [7] in teaching and encourages every location to build up a custom-tailored education program for themselves than can be constructed on the basis of concepts and the preparatory work of others.

Modification of knowledge and behavioral changes (second and third Kirkpatrick’s level) could be revealed by analyses of participants’ roles matrixes and the results from the alumni survey. This showed a considerably improved expertise in educational research and teaching quality as well as successful raising third-party funds at the present time compared to the beginning of the studies. 

The institutional support of participants has proven to be crucial for the successful completion of MME requirements. Protected time is required for the attendance phases in the different education locations, as well as for preparation and post-processing of courses. As Gusic et al. [33] showed, it was vital that the tasks accomplished by participants were of direct use to the home faculties. While all participants report strong personal development and professionalization, the institutional impact of the program demands a differentiated approach. On the one hand, the MME qualification is required for many faculty management positions and the chairs are staffed by MME graduates. Moreover, joint research projects are successfully initiated and MME master’s theses contribute to a significant rise in medical education publications in Germany [34]. All this represents the enormous impact of the MME program on the medical training landscape (Kirkpatrick’s fourth level). However, there are still clear institutional deficits, as shown by the fact that half of participants rate institutional support for teaching research in terms of human, financial and time resources as inadequate and that their positions do not match their qualification.

Here, it became clear that there is still a lack of transparent budgets and career paths. It also became evident that there are too few efforts to embed medical education research at the faculties themselves. The option of support from large funding institutions like the German Research Funding Organization (DFG) and the Federal Ministry of Education and Research which are still very restricted, also contribute to that. Further action is needed to promote structural changes in medical education. While in 2003 the new Regulating Licenses to Practice Medicine provided a basis for revising the curricula, the Master Plan for Medical Studies 2020 [35] provides the political basis for a new national legislation for medical education in Germany. The central issues “promotion of practical relevance in medical studies” and “strengthening of physicians' communication skills” are addressed in the MME curriculum by introduction of national OSCEs, fostering of scientific competences, closer integration of basic biomedical sciences and clinical care, as well as by communication trainings together with simulated patients. Another important task for the future will be to continue to establish a network of medical education and to further support the career opportunities of medical teaching experts in Germany. Equally important, however, is the further implementation of the international perspective and the exchange with international MME programs. 

## Conclusion

Medical education, like every scholarly discipline, needs good standards and qualified specialists. Through establishing an interdisciplinary and interfaculty postgraduate MME program, a community of medical educators was established as the basis for professionalization of teaching and educational research at the medical faculties in the German speaking region. It becomes apparent that bundling national or regional initiatives in a clearly structured network can become a driving force for comprehensive improvement of professionalization of medical education on a large scale. This model can serve as an example to successfully support professionalization in medical education. 

## Funding

The MME Program was supported by 72 grants from the Stifterverband für die Deutsche Wissenschaft and Heinz Nixdorf Foundation. 

## Acknowledgements

Without the generous support of the Association of Benefactors and the Heinz Nixdorf Foundation and the initiative of the Medizinischer Fakultätentag (MFT), the establishment of the MME study program would not have been possible. For this reason, we would like to extend our special thanks to Dr. Ekkehard Winter, formerly with the Association of Benefactors, and to Bettina Jorzik, program manager for study reform and academic young talents with the Association of Benefactors as well as to Prof. Gebhard von Jagow, former MFT president, and to Prof. Hans-Günther Sonntag, dean emeritus at the Medical Faculty Heidelberg. We would also like to thank Prof. Sabine C. Herpertz, Prof. Andreas Draguhn, Prof. Franz Resch, Prof. Reinhard Putz and PD Dr. Roman Duelli, former and current members of the course’s steering team, as well as all lecturers involved and all MME participants who have contributed to the success of the MME course through their qualification and their commitment, and also Liane Fessler-Ásgeirsson for MME management and coordination.

## Competing interests

The authors declare that they have no competing interests. 

## Figures and Tables

**Table 1:**
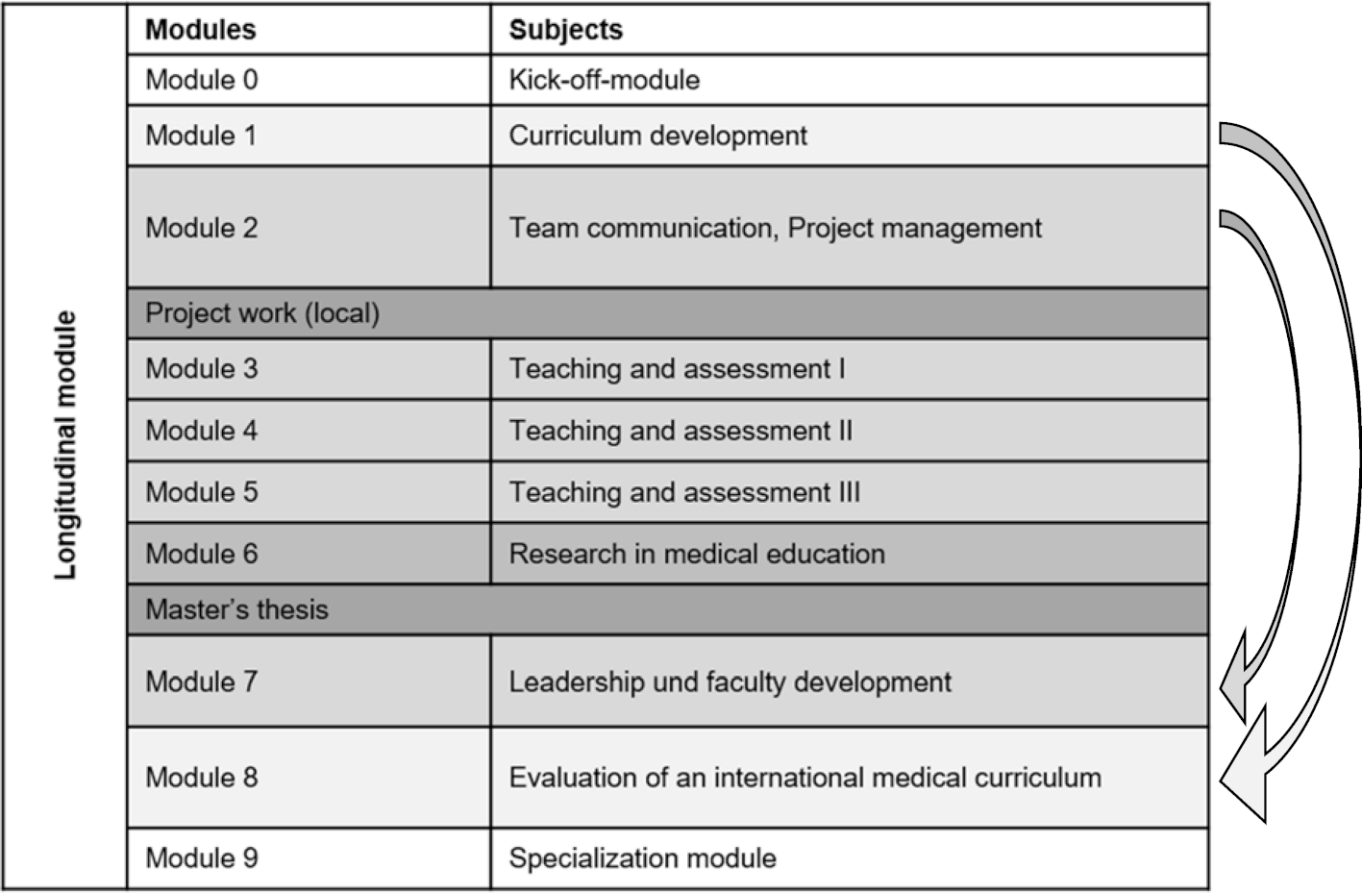
Content Overview of the MME Curriculum

**Table 2:**
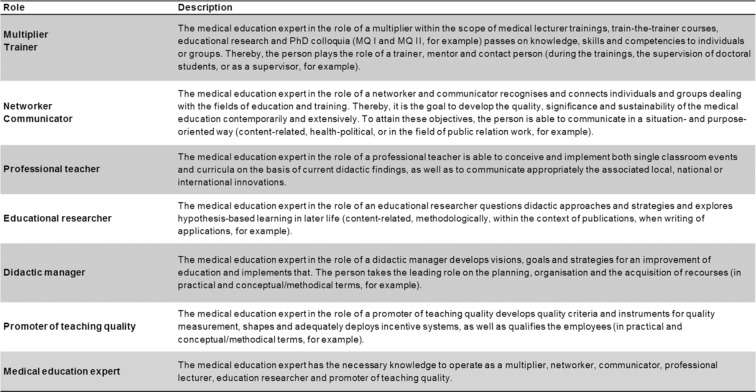
Defined roles for the MME roles matrix of self-evaluation
